# The Untapped Power of Soda Taxes: Incentivizing Consumers, Generating Revenue, and Altering Corporate Behavior

**DOI:** 10.15171/ijhpm.2017.69

**Published:** 2017-06-14

**Authors:** Sarah A. Roache, Lawrence O. Gostin

**Affiliations:** O’Neill Institute for National and Global Health Law, Law Center, Georgetown University, Washington, DC, USA.

**Keywords:** Public Health Law, Soda Taxes, Product Reformulation, Diet and Nutrition, Sugars

## Abstract

Globally, soda taxes are gaining momentum as powerful interventions to discourage sugar consumption and thereby reduce the growing burden of obesity and non-communicable diseases (NCDs). Evidence from early adopters including Mexico and Berkeley, California, confirms that soda taxes can disincentivize consumption through price increases and raise revenue to support government programs. The United Kingdom’s new graduated levy on sweetened beverages is yielding yet another powerful impact: soda manufacturers are reformulating their beverages to significantly reduce the sugar content. Product reformulation – whether incentivized or mandatory – helps reduce overconsumption of sugars at the societal level, moving away from the long-standing notion of individual responsibility in favor of collective strategies to promote health. But as a matter of health equity, soda product reformulation should occur globally, especially in low- and middleincome countries (LMICs), which are increasingly targeted as emerging markets for soda and junk food and are disproportionately impacted by NCDs. As global momentum for sugar reduction increases, governments and public health advocates should harness the power of soda taxes to tackle the economic, social, and informational drivers of soda consumption, driving improvements in food environments and the public’s health.

Soda taxes are a powerful, but underutilized, intervention to discourage sugar consumption and thereby reduce the burden of obesity and non-communicable diseases (NCDs).^1^ Despite major industry campaigns, cities and countries from Berkeley, California, to Barbados, are levying taxes on sugary beverages. Soda taxes incentivize consumers to purchase healthier products, while generating revenue to support health services and promotion programs. Beyond raising prices, advocacy and publicity surrounding “sin” taxes raise public awareness, sending a potent signal that consumers should beware before buying hazardous products.

The United Kingdom’s new levy on sugary beverages is yielding yet another powerful influence. Major UK soda manufacturers have announced plans to halve the sugar content of their beverages,^2^ bringing their products below the threshold for additional taxes. The experience in the United Kingdom presents an opportunity for governments and public health advocates to use taxes and other strategies to drive product reformulation among food and beverage companies. But as a matter of health equity, soda product reformulation should occur globally, especially in low- and middle-income countries (LMICs), which are disproportionately impacted by NCDs.

## The Health and Economic Impacts of Excess Sugar Consumption

Overweight and obesity, which have reached epidemic proportions worldwide, increase the risk of NCDs, including cancer, diabetes, and heart disease. In 2014, more than 1.9 billion adults, or 39%, were overweight or obese. Over 40 million children under 5 were overweight or obese.^3^ Globally, NCDs are the leading cause of death, killing around 40 million people each year.^4^

Although traditionally considered a problem for high-income countries, obesity and NCDs are increasing in LMICs. Almost half of the 40 million children who are overweight or obese live in Asia. Between 1990 and 2014, the number of African children who are overweight or obese increased from 5.4 million to 10.6 million.^3^ More than 85% of NCD deaths before the age of 70 occur in LMICs.^4^ In addition to the health impacts, obesity and NCDs impose significant financial burdens on households and national health systems, threatening the realization of development goals.^5^

Overconsumption of sugar – particularly in the form of sugary drinks – is a major contributor to the obesity epidemic.^6,7^ Worldwide, many populations far exceed the World Health Organization’s (WHO’s) recommendation to reduce free sugars to less than 10% of total daily energy intake.^8^ Sugary drinks are a particularly harmful source of added sugars because they provide no nutritional value other than energy and less satiation than other foods with similar calories. Once associated with the “Western diet,” sugary drinks are becoming increasingly popular in LMICs, with Latin America and Asia now leading the world in consumption.^9^

## Addressing the Economic, Informational, and Social Drivers of Consumption

In many societies, sugary drinks are heavily advertised, inexpensive, and widely available, driving consumption and delivering significant profits for industry.^10^ Well-designed taxes on sugary drinks reduce consumption from multiple angles, simultaneously tackling the economic, informational, and social drivers. Advocacy and publicity about taxes increase awareness of the health risks of sodas and de-normalize consumption. Price increases discourage purchases in favor of non-taxed options. Taxes, both directly, when absorbed by the manufacturer, or indirectly, when they result in decreased consumption, reduce corporate profits and incentivize product reformulation. Additionally, taxes generate revenue, which enables governments to adopt additional, complementary policies and programs to promote nutritious diets and physically active lifestyles (Figure). Subsidies for healthier food and beverage options complement taxes on unhealthy products by incentivizing healthier consumption patterns.^11^

**Figure:**
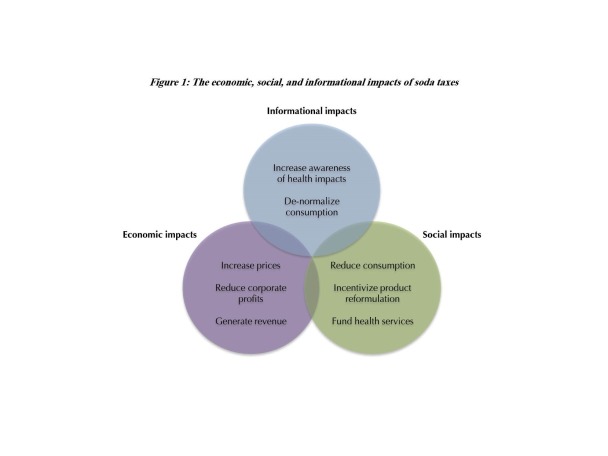
The Economic, Social, and Informational Impacts of Soda Taxes.

## Global Momentum for Soda Taxes: From a Trickle to a Stream

Globally, soda taxes are gaining momentum as effective and politically palpable ways to reduce consumption of added sugars. Since the start of this decade, a growing list of countries, including Barbados, Belgium, Chile, Dominica, France, Hungary, Kiribati, Mauritius, Mexico, and Tonga have enacted public health-based taxes on sugary beverages. By the end of 2016, 8 jurisdictions in the United States had adopted soda taxes, covering more than 8 million Americans.^12^ In October 2016, the WHO recommended that governments adopt excise taxes that raise retail prices of sugary beverages by at least 20%.^11^ While the WHO’s recommendation focuses on the potential of taxes to discourage consumption through price increases, revenue generation, consumer education, and product reformulation are recognized as co-benefits of well-designed taxation policies.

Evidence from early adopters shows that taxes can drive healthier consumption patterns. In 2014, Berkeley, California, became the first jurisdiction in the United States to implement a public health-based soda tax, levying a penny-per-ounce on sugary drinks. During the first year, consumption of sugary drinks in the city’s low-income neighborhoods fell by 21%.^13^ Following the introduction of Mexico’s 1-peso-per-liter sugary beverage tax in 2013, sales fell by 5.5% in the first year and 9.7% in the second year (compared to pre-tax sales figures), with the largest declines among low-socio-economic populations.^14^ Consistent with the public health objectives of the taxes, evidence from Berkeley^13^ and Mexico^14^ suggests that consumers may be substituting water for sugary drinks. Further research is warranted to determine the types of products consumers substitute for sugary drinks and the impact of substitution on longer-term health impacts of soda taxes.^15^

Soda taxes have potential for both positive and negative impacts on health and economic equality. Lower-income groups spend a greater percentage of their income to purchase taxed products than higher-income groups, meaning that taxes may increase economic ineqality.^16^ However, to the extent that soda taxes encourage reductions in consumption, they may lower expenditures on beverages. Additionally, soda taxes are more likely to yield greater health benefits among people in lower-income groups, who tend to be at greater risk of the health impacts of soda consumption and more responsive to increases in soda prices.^17^ Another potential adverse outcome of soda taxes is decreased fluid intake, particularly in settings with limited access to safe drinking water.^18^ While taxes can contribute to health equity by encouraging reduced consumption of unhealthy products among vulnerable populations, governments adopting soda taxes should provide subsidies or food aid to ensure that safe and nutritious alternatives are affordable and accessible.^11^

Part of the appeal of soda taxes is their capacity to generate revenue. In Berkeley, around $2 million of soda tax revenue has been allocated to programs designed to improve nutrition and decrease consumption of sugary drinks, including for the Berkeley Unified School District’s Cooking and Gardening Program^19^ and the Healthy Black Families’ Thirsty for Change! Program.^20^ The Mayor of Philadelphia promoted that city’s soda tax on the basis that revenue would fund popular community services such as universal preschool, libraries, and parks. In 2014, the Mexican soda tax generated approximately US$1.2 billion.^21^ Although the senate passed a resolution to use part of the proceeds to increase access to clean water in schools, it is unclear how the revenues have been spent.^22^ The WHO notes that transparency on the use of revenues is improved when governments earmark tax revenues for health promotion activities.^11^

## The UK’s New Soda Levy Drives Product Reformulation

The UK’s new tax on sugary beverages, due to take effect in April 2018, has revealed another benefit of soda taxes. The Soft Drinks Industry Levy aims to “help tackle childhood obesity by encouraging the reformulation of drinks to reduce levels of added sugar, as well as portion size reduction and the marketing of low sugar alternatives.”^23^ Soft drinks with total sugar content above 5 g/100 mL will be taxed at £0.18/L, while those with more than 8 g/100 mL will be taxed at £0.24/L.^24^ The levy will be payable by producers and importers. The UK levy is the first of its kind to include graduated tax rates based on total sugar content, which incentivizes the reduction of added sugar below 5 g/100 mL and the elimination of sugar to avoid the tax all together. The British government has committed to investing the revenue on increasing physical education and providing nutritious breakfasts in schools. The devolved administrations in Northern Ireland, Scotland, and Wales will determine how to spend their portion of revenues.^25^

Since the announcement of the levy in March 2016, the soft drink industry has criticized the policy and made commitments to reduce the sugar content of their products to avoid the higher rate. The British Soft Drinks Association, for example, has argued that soda taxes do not reduce obesity^26^ and negatively impact the economy. A report commissioned by the British Soft Drinks Association, which found that the levy would lead to 4000 job losses,^27^ has been criticized for underestimating the levy’s potential benefits and overestimating the costs.^28^ Meanwhile, Lucozade Ribena Suntory, the maker of two of the UK’s most popular sodas, announced it would cut sugar content in its beverages by an average of 50% and that all beverages will have less than 4.5 g of added sugar per 100 mL.^2^ Grocery chain Tesco^29^ and manufacturers Coca-Cola and PepsiCo also announced reformulation efforts to reduce added sugars.^30^ Originally, the UK government predicted that the tax would generate more than £520 million in the first year.^31^ Reductions in added sugars due to product reformulation efforts saw the government revise its estimate down to about £385 million.^32^

The UK’s sugary drinks tax appears to be the first to have sparked significant product reformulation commitments from multiple manufacturers. Although product reformulation in response to soda taxes (and more broadly) is relatively novel and has not been well studied, it offers a number of potential advantages. First, product reformulation does not rely solely on consumers changing their soda consumption habits in response to price increases. Instead, added sugars are reduced at the manufacturing level, resulting in less harmful products on supermarket shelves. Second, it does not rely on industry passing on the cost to consumers. In response to taxes on tobacco products, industry offered discounts or bulk pricing rather than passing on price increases intended to discourage consumption. Finally, as seen in the United Kingdom, product reformulation in response to soda taxes can drive competition among companies, both in terms of reducing sugar content to keep prices low and to offer healthier products.

## Product Reformulation Across the Globe: A Matter of Health Equity

The experience in the United Kingdom presents an opportunity for governments and public health advocates to use taxes and other strategies to drive product reformulation among food and beverage companies. As many more governments, including Australia, the Philippines, and India, actively consider taxing sugary drinks, it is pertinent to ask why UK manufacturers have committed to significantly reducing added sugars, and how similar reductions can be achieved in other countries.

Although the UK’s policy does not mandate reformulation, the government has taken a number of steps to encourage this response. Product reformulation is clearly stated as the primary objective in the explanatory notes to the legislation enacting the levy^23^ and the government’s public statements emphasize the focus of the tax on manufacturers and importers. The graduated tax structure offers a degree of flexibility for manufacturers, encouraging reductions in added sugars without requiring elimination. The tax applies nationally, bolstering the economic and practical cases for reformulation. Additionally, the government has provided ample time between announcing the tax and beginning collection, allowing manufacturers to plan for and execute reformulation strategies. While some time may be necessary, governments should be cautious that industry may use this argument to delay implementation and launch lobbying and litigation against taxes.

As a matter of health equity, soda product reformulation should not be restricted to higher-income countries. Reductions in added sugars should occur globally, especially in lower income countries, which are disproportionately impacted by NCDs.^33^ As public awareness of the health risks of soda consumption diminishes sales in high-income countries, companies are targeting emerging markets in Latin American, Asia, the Middle East, and Africa.^34^ While some LMICs may face barriers to implementing and enforcing graduated soda tax rates, flat excise taxes that raise the retail price of sugary drinks by 20% or more are likely to result in proportional reductions in consumption.^11,35^ In addition, governments and health advocates can point to reformulation efforts in the United Kingdom as evidence that reductions in sugar are practical for manufacturers and palatable among consumers. Including product reformulation as an aim of implementing legislation and publicizing global reformulation initiatives may also encourage local reformulation efforts across the globe.

The WHO can assist low- and middle-income governments to develop tax policies that encourage reformulation by sharing information and experiences, and providing technical assistance throughout adoption and implementation. Governments preparing to adopt soda taxes should identify and address potential challenges, such as ensuring access to alternative forms of hydration and providing sufficient resources for administration and collection. Early adopters such as the United Kingdom should contribute to the evidence base by establishing robust monitoring and evaluation processes to assess all impacts of its tax, including on price, sales, the sugar content of taxed products, and the use of tax revenues. It is also relevant to monitor the use and health impacts of replacement ingredients such as artificial sweeteners.

## Industry Opposition

One of the key challenges for all countries considering soda taxes is anticipating and addressing industry opposition. In some cities in the United States, where citizens vote on proposed soda taxes, industry has run well-coordinated and heavily resourced opposition campaigns. In San Francisco, for example, the American Beverage Association (ABA) spent $19 million opposing the city’s proposed tax.^36^ In many cases, the industry continues to fight against taxes even after they are adopted. In September 2016, plaintiffs including the ABA sued the City of Philadelphia, arguing that its soda tax violates the Pennsylvania Constitution. Philadelphia is committed to defending the suit, which is currently before a Pennsylvania appeals court.^37^

Mobilizing experts and grassroots advocates can counter some forms of industry opposition. In San Francisco, philanthropists and advocacy organizations provided $3.4 million in monetary and $6.1 million in nonmonetary support.^36^ In Boulder, Colorado, Healthy Boulder Kids bolstered public support prior to that city’s vote by educating the public on the health impacts of sugary drinks and local obesity rates. The celebrity chef, Jamie Oliver, has been a staunch advocate of the UK’s new tax, garnering over 150 000 signatures and successfully petitioning for a parliamentary debate.^38^ Of concern, multiple supporters of Mexico’s soda tax have received disturbing text messages including links laced with spyware. While soda companies deny involvement, the *New York Times* reports that the discovery “raises new questions about whether [spyware] tools are being used to advance the soda industry’s commercial interest in Mexico.”^39^

## Building on the Momentum of Soda Taxes to Reduce Diet-Related Disease

The global momentum in support of soda taxes confirms that many governments and societies seek effective and innovative means of further reducing diet-related disease. France, for example, recently banned refills of soda fountain drinks in public eateries, which complements its prohibition on vending machines in schools and its nation-wide soda tax.^40^ In 2012, the New York City Board of Health prohibited food service establishments from selling sugary drinks in containers larger than 16 ounces. While the New York Court of Appeals ultimately overturned the ban based on the board’s lack of authority,^41^ restrictions on serving sizes and packaging have the potential to further de-normalize and discourage consumption.^42^

Looking forward, governments might consider mandatory sugar reduction requirements for sodas and other foods and beverages. Argentina^43^ and South Africa^44^ have legally mandated maximum salt levels for a broad range of processed foods. This legally binding approach, which could be applied in the context of sugar reduction, offers the benefit of compulsory requirements on industry and penalties for non-compliance. However, implementation through law is typically more time consuming, requires broader political will, and effectiveness depends on adequate enforcement capacity.

## The Beginnings of a Global Movement Tackling Sugar Consumption

The global conversation around soda taxes is changing the way individuals, communities, governments, and companies are approaching sugar consumption. Perhaps the most profound change is industry’s significant reduction of added sugars in response to the UK’s new soda tax. Product reformulation – whether incentivized or mandatory – helps reduce overconsumption of sugars at the societal level, moving away from the long-standing notion of individual responsibility in favor of collective strategies to promote health. The epidemics of obesity and NCDs are a call to action for communities to demand healthier products, for companies to improve nutritional quality, and for governments to drive improvements in food environments, which are profoundly linked with health.

## Ethical issues

Not applicable.

## Competing interests

Authors declare that they have no competing interests.

## Authors’ contributions

Both authors made substantial contributions to the conceptualization of the topic, research and analysis, and to drafting the manuscript.
